# Short Lived Beauty

**Published:** 1891-04

**Authors:** 


### SHORT LIVED BEAUTY.

The woman who is pretty is far too liable to think that that is enough ; she will conquer her kingdom by means of it ; and when the day of reckoning, the day of fading comes, the kingdom will be hers by right of possession. Indeed she does not consider the day of fading ; it is something as difficult for her to realize as death itself is to the young ; it is far off, vague, all but impossible ; how is she ever going to look other than she does now, and still be herself ? And at any rate there are always the means to make the repairs of beauty, and sufficient unto the day is the evil thereof. And so, in an average of more than half the instances, she goes dancing off about her pleasure like a fly in the sun, as full of the present, as careless of the future ; she makes no preparation for the impending fate which is sure to come to her should she live long enough ; she relies on her face, her blushes, her dimples, her radiance, her smiles, her glances, her sweetness. To please, to attract, to marry, to marry well, is the mark she has set before her; and it does not need cultivation of the sterner virtues for that ; the sterner virtues are not greatly called into account in this quest, have little opportunity of asserting themselves, or even of being missed.

Nor is great intellectual cultivation in the scheme of our pretty woman's life ; according to her plan of action it is entirely unnecessary. Who cares for syllogisms, lectures, instructions, she unconsciously argues from rosy lips ? Who will stop to ask if the bright eyes have dulled themselves over dry pages of scholastic lore ? Let who will be learned ; it is enough for her to be gay and happy.

What, then, has our pretty creature left for the dim passages of middle age, when beauty has fallen away, but there still is left the desire to hold captive what once beauty gained ? The time is coming when there will be deep crescents round the mouth whose lovely curves have been dragged down by flaccid muscles, when there will be fine spider-web lines about the eyes, when there will be hollows in the cheeks, when the red and white of the skin will have become blurred and mottled or overlaid with yellow sallowness, when perhaps there will be present in the vacuous face only "that divine smile which has lost the two front teeth ?"

Let the pretty girl remember that in the darkness of that middle passage the beauty that she had before she entered it will not signify ; all faces are in the dark together then, the girl that was plain with the girl that was beautiful ; the wreck of beauty signifies then no more than the wreck of what never was beauty. It is the sweet voice, the kindly manner, the burden of what is said, the tender-heartedness of what is done, that tells with any effect then. It will not be long before she arrives at this time, which, in comparison to the blaze of youth, neighbors close on the dark ; and she will need them all with which she can have filled her intellect and fed her soul, all that wit and virtue and breeding can have given her, in order to retain any thing of that kingdom to which in the early days she felt herself born by right divine.—*Ex.*

